# Residual myocardial hyperemia in regadenoson stress/rest quantitative perfusion cardiac magnetic resonance

**DOI:** 10.1007/s11547-025-02062-3

**Published:** 2025-08-23

**Authors:** Gorka Bastarrika, Ana Ezponda, Javier Muñiz-Sáenz-Diez, Marta Vidorreta, Amaia Ochoa González, Juan José Gavira, Nahikari Salterain

**Affiliations:** 1 Department of Radiology, Clinica Universidad de Navarra, Pamplona, Spain; 2 Instituto de Investigación Sanitaria de Navarra (IdiSNA), Pamplona, Spain; 3 Department of Cardiology, Clínica Universidad de Navarra, Pamplona, Spain; 4 Siemens Healthineers, Madrid, Spain

**Keywords:** Cardiac magnetic resonance, Perfusion, Vasodilator, Coronary artery disease

## Abstract

**Purpose:**

This study sought to investigate the presence of residual myocardial hyperemia on the recovery phase in patients undergoing stress CMR.

**Material and methods:**

Fifty patients with clinical indication for stress CMR underwent quantitative perfusion imaging in resting conditions, after regadenoson-induced hyperemia (400 mcg, 5 mL), and 10 min after recovery with euphylline. Studies showing hypoperfusion due to ischemia and/or prior myocardial infarction were excluded. Global myocardial blood flow during rest (MBF_rest_), stress (MBF_stress_) and recovery (MBF_recovery_) and MPR indices (MPR_stress/rest_ and MPR_stress/recovery_) were calculated using automated pixel-wise quantitative myocardial perfusion mapping.

**Results:**

A total of 30 patients (22 males, mean age of 62.7 ± 1 years) were included in the analysis. Global MBF_rest_ and MBF_stress_ were 0.83 ± 0.2 mL/g/min and 2.1 ± 0.6 mL/g/min, respectively. After recovery with euphylline, myocardial perfusion did not return to the resting values (MBF_recovery_ of 0.92 ± 0.3 mL/g/min) and statistically differed from MBF_rest_ (*p* < 0.01), suggesting residual myocardial hyperemia. This resulted in an abnormally low MPR_stress/recovery_ (2.43 ± 0.7) with respect to MPR_stress/rest_ (2.56 ± 0.7) (*p* = 0.03). A linear mixed-effects model accounting for repeated measures revealed statistically significant group differences over time in global MBF (mean difference 0.1, 95% CI 0.02–0.17, *p* = 0.01) and global MPR (mean difference −0.13, 95% CI −0.25 to −0.02, *p* = 0.02).

**Conclusion:**

Despite the use of euphylline to counteract the vasodilator effect, MBF does not completely revert to resting values and MBF_recovery_ cannot be used as a substitute for MBF_rest_ when regadenoson is used. Consequently, a rest/stress protocol is advised for quantitative CMR perfusion to obtain accurate MBF and MPR parameters.

**Supplementary Information:**

The online version contains supplementary material available at 10.1007/s11547-025-02062-3.

## Introduction

Stress-perfusion cardiac magnetic resonance (CMR) imaging has become a core component of the evaluation of symptomatic individuals with known or suspected coronary artery disease (CAD) [1]. Several studies have shown that this modality is highly accurate in diagnosing CAD [2, 3] exhibiting similar performance to the established reference standard for evaluating vessel-specific ischemia (fractional flow reserve, FFR) in terms of outcomes [4].

Stress-perfusion CMR is generally performed with vasodilator drugs [5]. The European Medicines Agency (EMA) has recently approved the clinical use of regadenoson for myocardial perfusion imaging in patients unable to undergo adequate exercise stress. This medication is a highly selective A2A adenosine receptor agonist that possesses a longer half-life compared to other vasodilators, such as adenosine, dipyridamole, or adenosine triphosphate (ATP). Aminophylline can be utilized to reverse the effect of regadenoson, although there is concern regarding the potential persistence of residual myocardial hyperemia despite its administration [6]. Consequently, resting perfusion values of CMR examinations performed with a conventional stress/rest perfusion protocol could be potentially affected. This is of particular interest when newly developed quantitative perfusion CMR is considered, which allows more comprehensive analysis of the myocardial perfusion by quantitative calculation of the myocardial blood flow (MBF) [7].

This study aimed to explore the persistence of residual myocardial hyperemia during the recovery phase in patients undergoing quantitative stress-perfusion CMR with regadenoson following a conventional stress/rest perfusion scheme. If demonstrated, modification of the protocol to rest/stress perfusion would be advised.

## Materials and methods

### Study population

Data from 50 patients with known or suspected coronary artery disease who underwent clinically indicated stress-perfusion CMR examination between May and October 2022, were retrospectively analyzed. The studies were conducted on individuals who were hemodynamically stable (i.e., with adequate blood pressure, cardiac output, and tissue perfusion without signs of ischemia, shock or end-organ dysfunction), had normal glomerular filtration rates (GFR > 60 mL/min/1.73 m^2^), and did not have contraindications for the administration of regadenoson. All patients were instructed to refrain from consuming caffeine-containing substances for 24 h prior to the examination, with compliance verified by a nurse before performing the procedure. They were also advised to take their regular medications on the day of the study, including beta-blockers and nitrates.

### CMR protocol

**Fig. 1:**
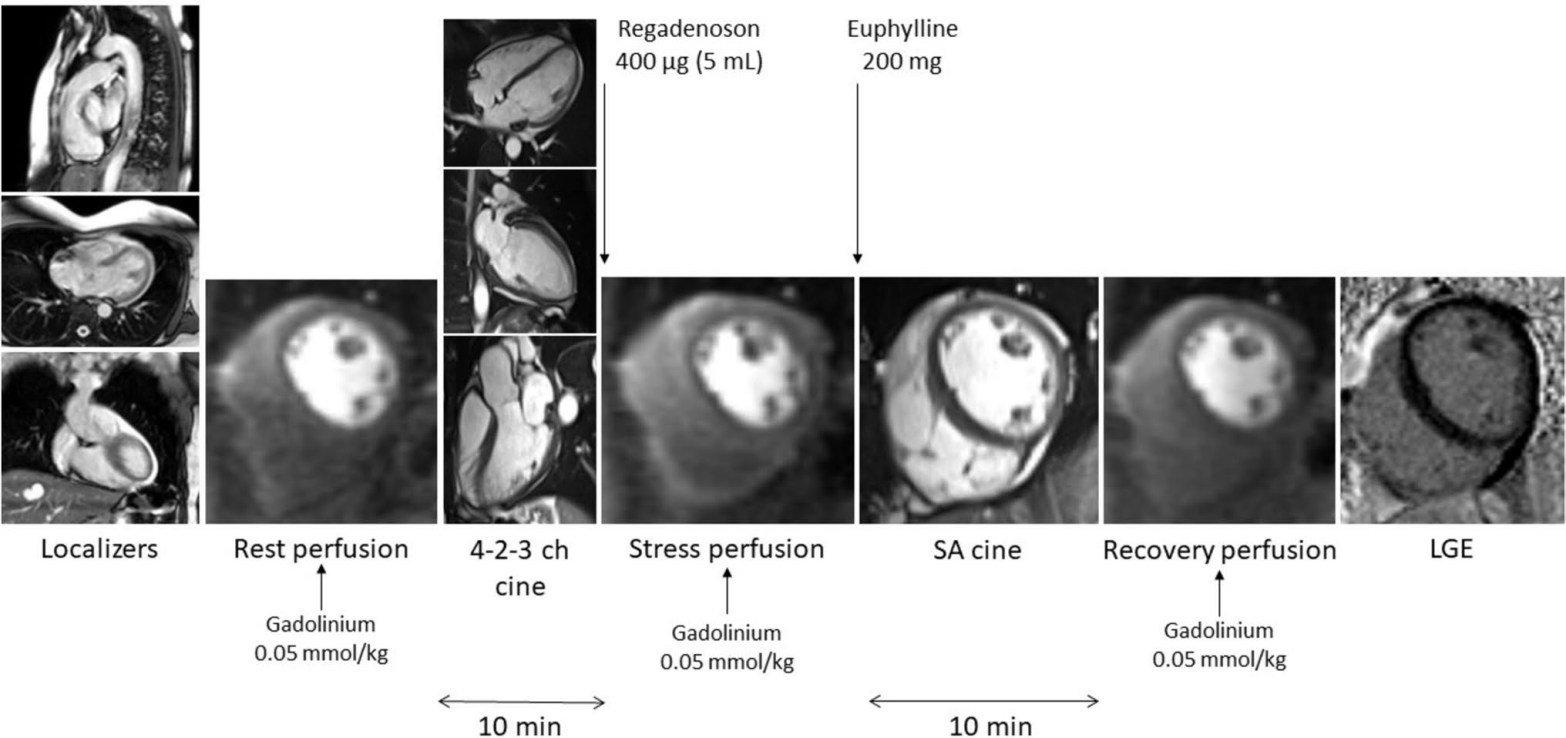
CMR protocol

CMR examinations were performed on a 1.5-Tesla scanner (Magnetom Aera, Siemens Healthineers, Erlangen, Germany). The study protocol included long- and short-axis steady-state free precession (SSFP) cines, first-pass myocardial perfusion imaging during (1) resting conditions (rest), (2) 80 s after the intravenous administration of regadenoson (Rapiscan, GE Healthcare AS) at a fixed dose of 0.4 mg (5 ml) (stress), and (3) during post-euphylline recovery phase, (recovery) each separated by 10 min, and late gadolinium enhancement (LGE) images (Fig. 1). For perfusion imaging, three slices representative of the basal, mid, and apical segments of the left ventricle were obtained using a saturation recovery (SR) dual-sequence method (simultaneous low-resolution arterial input function and a high-resolution myocardial perfusion acquisitions) with a multi-slice, free-breathing, saturation recovery pulse sequence with fast low-angle shot (FLASH) readout, acquired over 60 heartbeats [8]. Sequence parameters were as follows: TE 1.0 ms, TR 2.1 ms, flip angle 14°, bandwidth 1085 Hz/pixel, matrix 192 × 111 (1.9 × 2.4 mm^2^), field of view 360 × 270 × 8 mm^3^, parallel imaging TPAT3, saturation time (TS) 100 ms, trigger delay (TD) 62 ms, imaging duration of 59 ms, total duration of 143 ms/slice. The recovery phase was consistently obtained 10 min after the administration of regadenoson to avoid variability. Euphylline (anhydrous theophylline, 200 mg iv. diluted in 50 ml of saline) was used to revert the vasodilator effect of regadenoson in all patients. A total dose of 0.15 mmol/Kg of gadobutrol (Gadovist, Bayer AG, Berlin, Germany) was administered at a flow rate of 4 ml/s, with a double-head injector (0.05 mmol/Kg for each perfusion examination) [9]. Heart rate and arterial blood pressure were continuously monitored.

#### Patient monitoring

Hemodynamic response to regadenoson was determined during rest, stress and recovery conditions by measuring heart rate (HR) changes. Blood pressure (BP) values were obtained during rest and stress conditions. Rest HR and BP values were registered before regadenoson administration. Stress HR was defined as the highest HR during stress perfusion, whereas stress BP was taken just after the stress first-pass myocardial perfusion scan. Recovery HR was registered 10 min after euphylline administration.

#### CMR image analysis

**Fig. 2:**
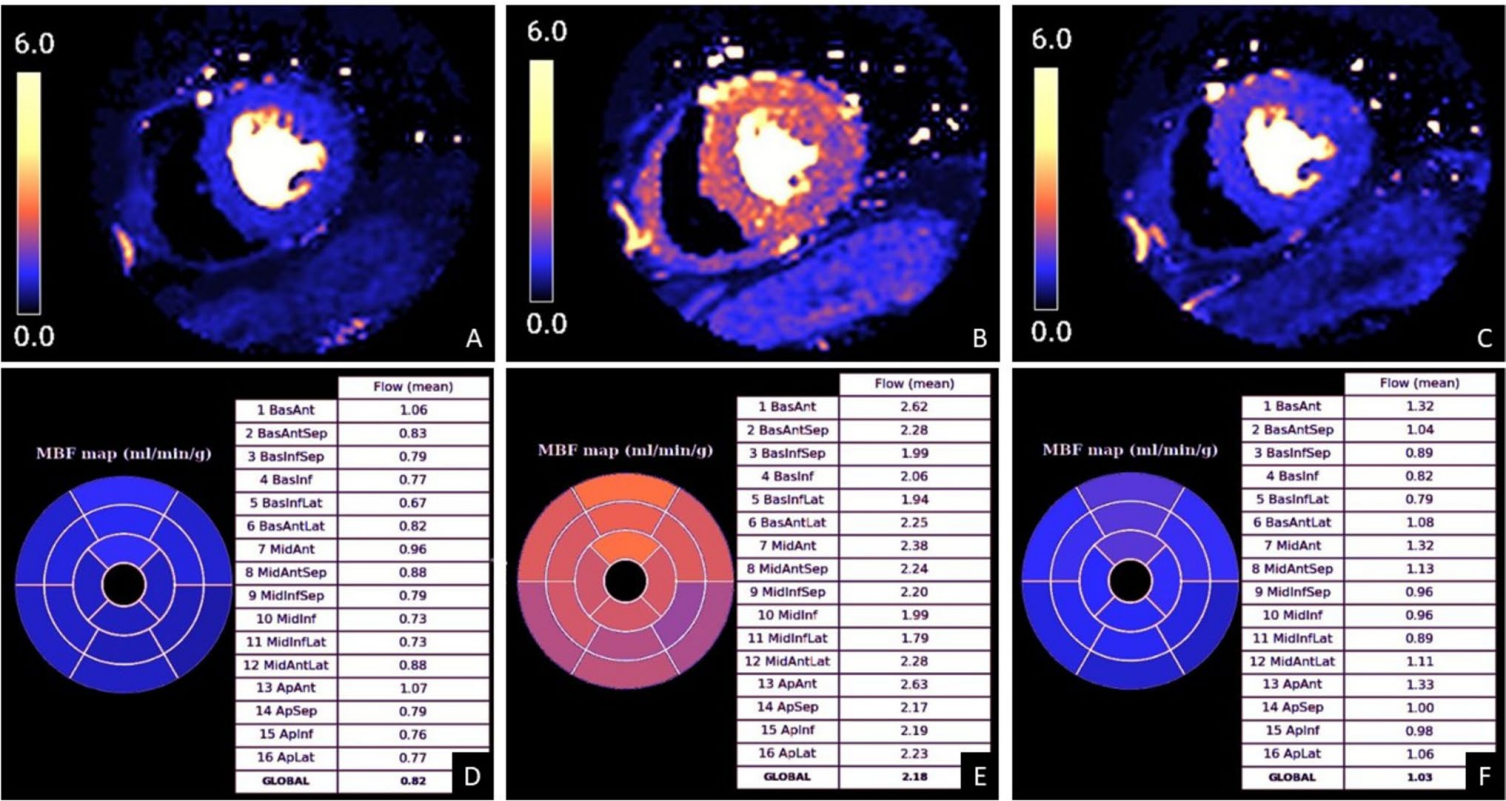
Representative example of color-coded perfusion maps with respective quantitative myocardial blood flow (MBF) values. A, B, C. Mid-ventricular color-coded perfusion maps at rest (**A**), stress (**B**) and recovery (**C**). Quantitative myocardial blood flow (MBF) values at rest (**D**), stress (**E**) and recovery (**F**). Note slightly higher MBF values at recovery compared to rest, indicating persistent vasodilator effect of regadenoson

Left ventricular volumes, function and mass were calculated on short-axis cine slices using dedicated commercial software (cmr 42, Circle Cardiovascular Imaging Inc., Calgary, Canada) [10]. Quantitative analysis of perfusion data is detailed elsewhere [11]. Briefly, first-pass perfusion images were initially corrected for motion and for surface coil intensity variation. Surface coil variation was corrected with proton density (PD)-weighted images that were acquired at the start of the scan, whereas motion was corrected using non-rigid image registration implemented within the Gadgetron software framework [12]. Signal intensity data were converted to gadolinium concentration, and MBF was calculated on a pixel-wise basis by blood tissue exchange constrained deconvolution model. Pixel-wise flow values were calculated using assumed or default T1 values for blood, rather than being individually corrected for hematocrit in each patient (Fig. 2). Based on an artificial intelligence tool, the American Heart Association (AHA) segmentation model was applied to automatically calculate segmental MBF as an average of all pixels. Values for each of the AHA 16 segments were recorded for rest, stress, and recovery (MBF_stress_, MBF_rest_, MBF_recovery_). MPR was calculated as the ratio of MBF_stress_ to MBF_rest_ (MPR_stress/rest_) and the ratio of MBF_stress_ to MBF_recovery_ (MPR_stress/recovery_). Automatically contoured perfusion maps were visually assessed for quality control. For the study purpose, segments with significant artifact or hypoperfusion due to ischemia and/or prior myocardial infarction (as demonstrated on LGE images as subendocardial or transmural enhancement) were excluded from the analysis. Presence and extent of LGE were visually assessed. The readers performed image analysis blinded to the clinical data, the timing of the perfusion CMR acquisition (rest, stress, or recovery), other CMR parameters, or the quantitative CMR perfusion values.

#### Statistical analysis

Data are described as mean ± standard deviation or as frequencies and percentages, as appropriate. Normal distribution of data was assessed with the Kolmogorov–Smirnov test. Intraindividual comparison of MBF_rest_ and MBF_recovery_ and MPR_stress/rest_ and MPR_stress/recovery_ was performed using the paired-samples T test. In a secondary analysis, we used a linear mixed-effects models with random intercepts by subject to account for within-subject correlations from repeated measures, without averaging segmental values. For the mixed model, we designated the patients as a random effect (random intercept), while the time was treated as fixed effect [13]). SPSS (version 29.0 / SPSS Inc., Chicago, IL) and Stata (version 15.0, College Station, TX, USA) were used for the statistical analysis. A *p* value < 0.05 was considered statistically significant.

## Results

### Patient characteristics

**Fig. 3:**
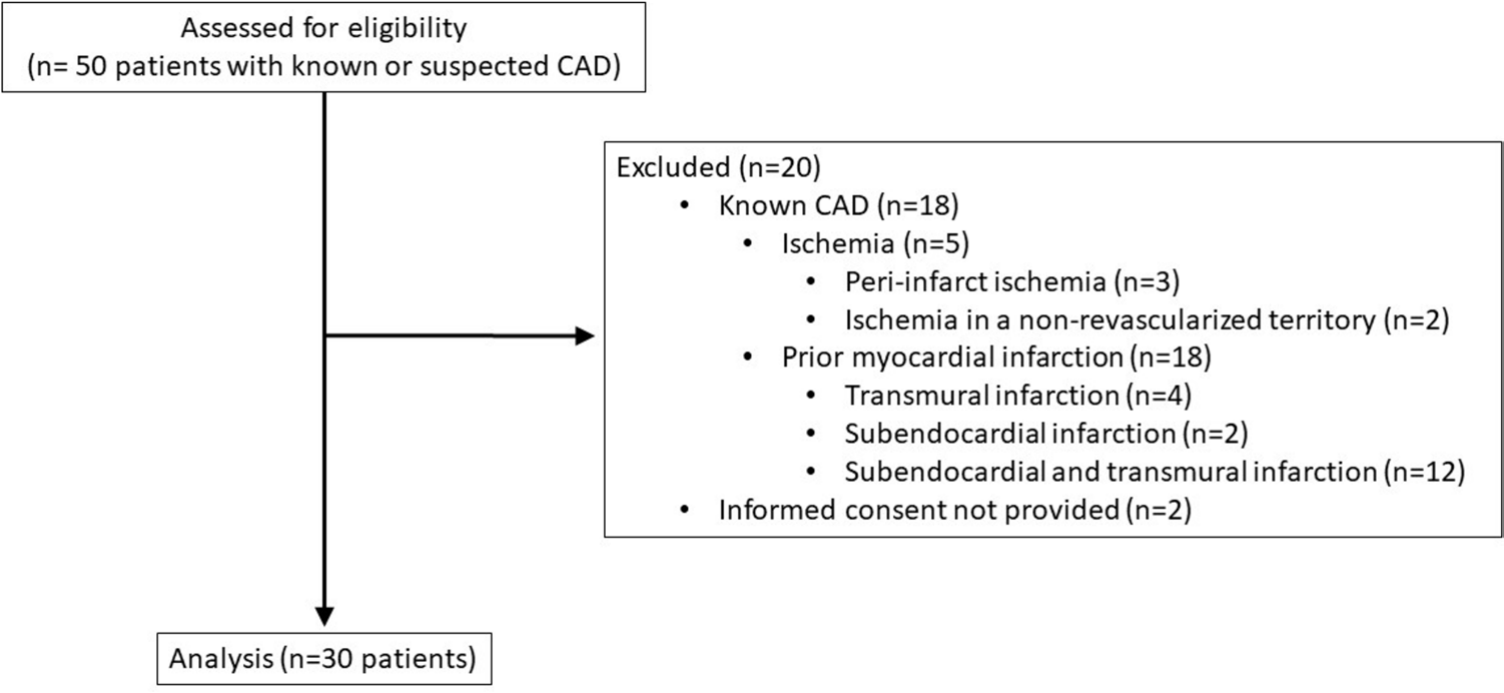
Flowchart of participants according to the applied inclusion and exclusion criteria. CAD: coronary artery disease

All individuals underwent stress-CMR examinations without any adverse events or complications. Among the initially recruited cohort of 50 individuals, 18 showed hypoperfusion resulting from ischemia and/or prior myocardial infarction and, thus, were excluded from the analysis. Further retrospective review of medical records revealed that 2 patients did not provide consent to be included in any research study. Consequently, a total of 30 subjects were included in the final analysis (Fig. 3).

Patient demographics, cardiovascular risk factors, and clinical indications for stress-CMR examinations are shown in Table 1. Most patients were male (73.3%), with a mean age of 62.7 ± 1 years, a mean heart rate of 67.5 ± 12.8 beats per minute (bpm), and a mean body surface area (BSA) of 1.9 ± 0.2 m^2^. At the time of examination, most patients were in sinus rhythm (90%).

**Table 1:** Patient demographics, cardiovascular risk factors, and clinical indications for stress CMR

Variables	n
Gender (male/female)	22/8
Age (years)	62.7 ± 1
Weight (kg)	81.5 ± 18.8
BMI (kg/m^2^)	27.6 ± 5
BSA (m^2^)	1.9 ± 0.2
Mean heart rate (bpm)	67.5 ± 12.8
*Cardiac rhythm*	
- Sinus rhythm	27
- 1st degree heart block	1
- Atrial fibrillation	1
- Pacemaker	1
*Cardiovascular risk factors*	
- Smoker/non-smoker/former smoker	6/12/12 (20%/40%/40%)
- Systemic hypertension	15 (50%)
- Dyslipidemia	21 (70%)
- Diabetes mellitus	13 (43.3%)
- COPD	1 (3.3%)
- Hyperuricemia	4 (13.3%)
- Family history of CAD	10 (33.3%)
*Prior revascularization*	
- Bypass	3 (10%)
- Stent	2 (6.7%)
*Clinical indication for stress CMR*	
- Cardiomyopathy	10 (33.3%)
- Angina/atypical chest pain	9 (30%)
- Prior revascularization with chest pain	5 (16.7%)
- Dyspnea	3 (10%)
- New onset heart failure	3 (10%)

BMI, Body mass index; BSA, Body surface area; bpm, beats per minute; COPD, chronic obstructive pulmonary disease; CAD, coronary artery disease; CMR, cardiac magnetic resonance. Data are presented as mean ± standard deviation or as frequencies and percentages, as appropriate

Cardiovascular risk factors were observed in most individuals, the most prevalent being dyslipidemia and systemic hypertension. Stress CMR was conducted in 10 patients suspected of cardiomyopathy, 9 subjects with angina/atypical chest pain, 5 patients with a history of revascularization and chest pain, 3 patients experiencing dyspnea and 3 individuals presenting with new-onset heart failure.

### CMR findings

The mean left ventricular ejection fraction, volumes and mass were within normal limits. Normal left ventricular morphology was observed in most patients (56.7%), with concentric remodeling being the most frequent abnormal finding (20%). Late gadolinium enhancement was absent in 60% of patients. Mesocardial and subepicardial LGE pattern were demonstrated in 30% and 6.7% of subjects, respectively, indicative of myocardial fibrosis. Among patients, 43.3% had a normal CMR examination, while hypertensive cardiomyopathy was diagnosed in 26.7%, myocarditis in 16.7% and dilated cardiomyopathy in 13.3%. Further details of CMR findings are outlined in Table 2.

**Table 2:** CMR findings

LV-EF (%)	65.6 ± 13.1
BSA-indexed LV-EDV (mL/ m^2^)	76.3 ± 26.9
BSA-indexed LV-ESV (mL/ m^2^)	28.6 ± 23.1
BSA-indexed LV-mass (g/ m^2^)	63.2 ± 16.6
*Left ventricular morphology*	
- Normal	17 (56.7%)
- Concentric remodeling	6 (20%)
- Dilated	3 (10%)
- Eccentric remodeling	3 (10%)
- Hypertrophic	1 (3.3%)
*Late gadolinium enhancement pattern*	
- Absent	18 (60%)
- Mesocardial	9 (30%)
- Subepicardial	2 (6.7%)
- Insertion points	1 (3.3%)
*CMR diagnosis*	
- Normal	13 (43.3%)
- Hypertensive cardiomyopathy	8 (26.7%)
- Myocarditis	5 (16.7%)
- Dilated cardiomyopathy	4 (13.3%)

LV, left ventricle; EF, ejection fraction; BSA, body surface area; EDV, end-diastolic volume; ESV, end-systolic volume; CMR, cardiac magnetic resonance

### Hemodynamic response to regadenoson

Heart rate and blood pressure changes across rest, stress, and recovery phases are shown in Table 3. There was an overall increase of 42.2% of heart rate (mean difference of 26.7 ± 15.8 bpm, *p* < 0.001) from rest to stress and a decrease of 25.5% (mean difference of 25 ± 1 bpm, *p* < 0.001) from stress to recovery. Resting heart rate and recovery heart rate did not statistically differ (mean difference of 1.7 ± 10.1 bpm, *p* = 0.19). Systolic blood pressure and diastolic blood pressure decreased from rest to stress (mean of 8.4 ± 14.5 mmHg and 7.7 ± 10.8 mmHg, respectively, *p* < 0.05).

**Table 3:** Heart rate and blood pressure changes during rest, stress, and recovery

	Rest	Stress	Recovery
Mean HR	67.5 ± 12.8	94.1 ± 16.4	69.1 ± 12.6
Systolic BP	135.1 ± 18.6	126.7 ± 22.6	
Diastolic BP	63.6 ± 11.3	55.9 ± 12.7	

Notes. HR: heart rate; BP: blood pressure

### Myocardial blood flow and myocardial perfusion reserve

**Fig. 4:**
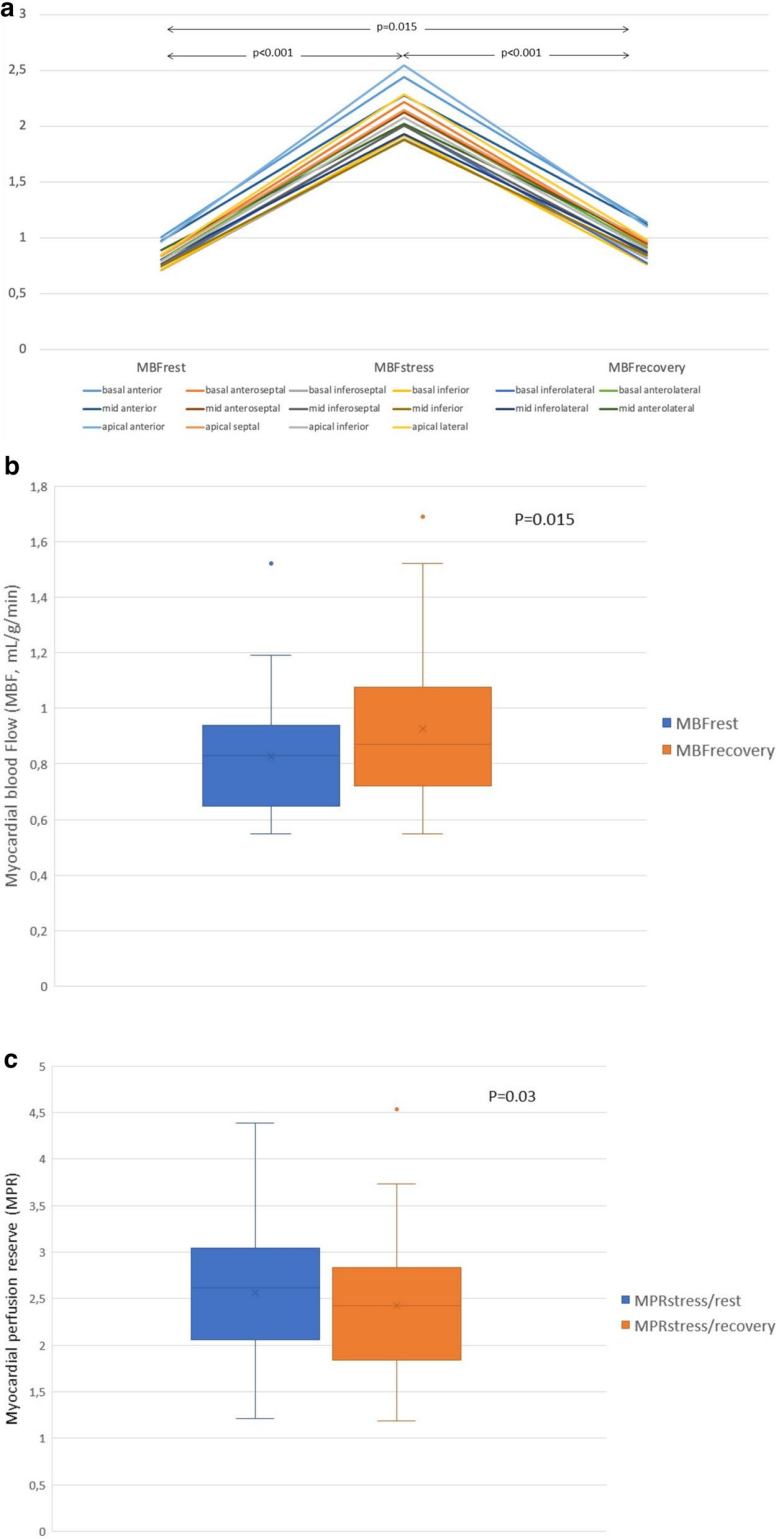
Differences in myocardial blood flow (MBF) and myocardial perfusion reserve (MPR) during rest, stress and recovery. **A**. Changes in myocardial blood flow (MBF) estimates during rest, stress, and recovery of each myocardial segment. **B** Boxplots with summary statistics of global MBF at rest and recovery. **C**. Boxplots with summary statistics of global MPR at rest and recovery

Myocardial blood flow (MBF) was estimated in all 480 left ventricular myocardial segments. No segments were excluded due to artifact. The global MBF per patient was calculated as the mean of the MBF values across myocardial segments. Changes in MBF values during rest, stress, and the recovery phases for each myocardial segment are shown in Fig. 4. There was statistically significant difference between.

Global MBF_rest_ and global MBF_stress_ (0.83 ± 0.2 mL/g/min vs. 2.1 ± 0.6 mL/g/min, 95% CI − 1.49 to − 1.06, *p* < 0.001) and between MBF_stress_ and global MBF_recovery_ (2.1 ± 0.6 mL/g/min vs. 0.92 ± 0.3 mL/g/min, 95% CI 0.96 to 1.39, *p* < 0.001)). Global MBF_recovery_ values were higher than MBF_rest_ values. This difference was statistically significant (mean difference of − 0.1, 95% CI − 0.17 to − 0.02, *p* = 0.015). Similarly, the ratio between MBF_stress_ and MBF_rest_ was higher (mean MPR_stress/rest_ of 2.56 ± 0.7) as compared with the ratio between MBF_stress_ and MBF_recovery_ (mean MPR_stress/recovery_ of 2.43 ± 0.7) with a mean difference of 0.13 ± 0.3 (95% CI 0.01–0.25, *p* = 0.03). A summary of the comparison between global rest and recovery MBF and MPR measurements is shown in Table 4 (Supplemental Table 1).

**Table 4:** Comparison of global rest and recovery myocardial blood flow (MBF) and myocardial perfusion reserve (MPR) measurements

	Mean ± SD	Mean difference	Cohen’s d	95% CI	*p*
MBF_rest_	0.83 ± 0.2	− 0.1 ± 0.2	0.5	− 0.17 to − 0.02	0.015
MBF_recovery_	0.92 ± 0.3				
MPR_stress/rest_	2.56 ± 0.7	0.13 ± 0.3	0.43	0.01 to 0.25	0.03
MPR_stress/recovery_	2.43 ± 0.7				

CI, confidence interval; *p* value < 0.05 was considered statistically significant

We applied a linear mixed-effects model to appropriately account for within-subject correlation due to repeated measures over time. Repeated measures within subjects of MBF and MPR per myocardial segment are shown in supplemental Table 2. The marginal means of group differences at each time points' test revealed statistically significant differences between groups when global MBF (mean difference of 0.1, 95% CI 0.02–0.17, *p* = 0.01) and global MPR (mean difference of − 0.13, 95% CI − 0.25 to 0.02, *p* = 0.02) were estimated.

### Adequacy of the sample size

A post hoc power analysis was conducted to assess the adequacy of the sample size for detecting the observed difference in global MBF between rest and recovery. Based on the observed mean difference (0.098), the estimated standard deviation of the paired differences (0.295), the empirical correlation between visits (r = 0.6698), and a total of 30 participants, the estimated power was 58% for a two-sided test at α = 0.05.

## Discussion

This quantitative stress-perfusion CMR study demonstrates that myocardial blood flow values at rest and during the recovery period statistically differ when regadenoson is used as a vasodilator. Consequently, there is also a difference in the myocardial perfusion reserve, which is slightly underestimated if a stress/rest perfusion protocol is used. To our knowledge, this is the first study to provide evidence of regadenoson kinetics using absolute myocardial flow quantification.

A conventional stress-perfusion CMR protocol comprehends initial first-pass myocardial perfusion imaging under vasodilator-induced stress followed by resting perfusion, usually 10–15 min later [1]. This is generally performed using adenosine or, more recently, regadenoson. Both induce equivalent vasodilator effect [14] but the latter seems to be better tolerated than adenosine due to its selective binding of adenosine 2A receptors [15]. Regadenoson seems to achieve, however, a prolonged low level of coronary vasodilation [16], irrespective of the use of an adenosine receptor antagonists like euphylline. Our study demonstrates that MBF values during rest and the recovery phases are not equivalent. The persistent myocardial hyperemia induced by regadenoson leads to abnormally high MBF values during recovery and results in an underestimation of MPR. This observation aligns with findings from a small experimental investigation [14] and a study involving healthy volunteers [15]. One possible explanation is the low affinity of aminophylline for the A2A receptor, which permits circulating regadenoson to re-bind to this specific receptor after stress [15].

Several intravenous contrast injection strategies have been considered for myocardial perfusion imaging. Traditionally, stress-perfusion CMR has used 50–100% higher contrast doses than other cardiac indications [17]. The rationale for using 0.05 mmol/kg per perfusion in this study was based on the recent GadaCAD trials that showed high diagnostic accuracy of gadobutrol-enhanced CMR (0.1 mmol/kg) to assess myocardial perfusion and LGE in adult patients with known or suspected CAD and in the fact that the published data do not support a dose-related tendency for higher sensitivity for stress-perfusion CMR [9].

On the other hand, the clinical assessment of stress-perfusion CMR typically relies on visual interpretation or, less frequently, on semiquantitative calculations derived from the perfusion time-signal intensity curves obtained during myocardial hyperemia and resting conditions [5, 20]. Recently introduced fully automated quantitative techniques involve measuring arterial input and myocardial tissue functions to objectively determine myocardial blood flow. In our study, we utilized a specific dual-sequence method [8]. This technique has shown to allow separate optimization of imaging parameters for blood and myocardial tissue, resulting in accurate AIF measurements and reliable perfusion mapping with low variability in healthy subjects. Further, the dual-sequence method simplifies the imaging protocol by eliminating the need for two separate contrast injections, as required in the dual-bolus technique. This reduces the complexity of the procedure and potential for errors related to contrast administration. Additionally, the dual-sequence method can be integrated with automated image processing workflows, further enhancing its clinical feasibility. It is interesting to note, however, that the obtained myocardial perfusion values may differ depending on the sequence. In a recent retrospective observational study conducted by Chong et al. [18], stress CMR was performed using both dual-sequence and dual-bolus techniques on the same day. The study found that while there was a very good correlation between MBF and MPR values, there were significant differences in global stress and MBF_rest_ values between the two methods. This underlines the need of further investigation to clarify the most appropriate approach for myocardial perfusion quantification.

This study provides evidence that the order of the study protocol should be modified with regadenoson, especially if accurate quantitative analysis of myocardial perfusion is required. A rest/stress-perfusion protocol would yield more reliable results compared to a conventional stress/rest perfusion protocol, as it provides true resting MBF values instead of abnormally high MBF values resulting from persistent myocardial hyperemia during the recovery period. In this study, we employed euphylline to revert the vasodilatory effects of regadenoson. Alternative reversal agents that have been shown to be effective include intravenous theophylline (400 mg in 500 mL bag) and oral (caffeinated beverage) or intravenous caffeine (caffeine citrate, 60 mg in 3 mL vial) [19]. Interestingly, we observed markedly abnormal MBF values during the recovery phase 10 min following administration of euphylline. While extending the waiting period may yield different MBF measurements, this approach is generally impractical, as it would substantially increase the duration of an already long examination, negatively affecting patient throughput or scanner availability. As an alternative, a stress-only imaging protocol may be considered. This strategy would allow for shorter scan times and reduced contrast agent exposure by omitting the rest perfusion. However, this approach precludes the calculation of MPR values, which have been proven to reduce interpretative subjectivity compared to visual analysis [20] and to provide greater diagnostic accuracy than stress MBF alone by identifying ischemic burden in multivessel CAD, differentiating it from microvascular disease and providing a strong, independent predictor of adverse cardiovascular outcomes [11, 21, 22]. Therefore, quantitative myocardial perfusion assessment is likely to offer significant clinical benefit for patients with multivessel CAD, balanced ischemia, or microvascular disease-populations at elevated risk for heart failure, myocardial infarction, and sudden cardiac death due to extensive underlying coronary pathology, in whom conventional stress testing may yield false-negative results owing to diffuse and uniform perfusion deficits [12, 23]. This study is not without limitations. Firstly, the sample size is small, and there is a lack of a validation population. Nonetheless, we observed significant differences during the recovery phase in comparison with true resting perfusion values, which is noteworthy given the expanding use of regadenoson. Whether this difference is clinically relevant must be explored in further research. Secondly, although our investigation was conducted in a clinical setting, patients with hypoperfusion due to ischemia and/or prior myocardial infarction were excluded from the analysis for the study’s purpose. While this allowed us to characterize myocardial perfusion without confounding factors, further research should examine whether our findings hold true in patients with myocardial ischemia or established myocardial infarction. Thirdly, the use of several contrast bolus injections may influence the myocardial signal intensity and perfusion values. To avoid this, the sequence has been developed to provide signal intensity conversion to contrast agent concentration and only at this stage are quantification models applied to the perfusion data to calculate MBF [23]. Finally, we utilized a research sequence for quantitative CMR perfusion using a specific vendor platform and AI tool. Despite several investigations demonstrating its potential [22, 24, 25], its impact on routine clinical practice remains to be determined. Further, potential variation in reproducibility on different platforms may be expected.

In conclusion, in stress-perfusion CMR, even with the administration of euphylline to counteract the vasodilator effect, MBF does not completely revert to resting values and MBF_recovery_ cannot be used as a substitute for MBF_rest_ when regadenoson is employed as vasodilator. Therefore, for obtaining precise MBF and MPR parameters in quantitative CMR perfusion, it is recommended to use a rest/stress protocol with regadenoson. Further investigation is needed to validate the clinical significance of these findings across different clinical scenarios.

## Supplementary Information

Below is the link to the electronic supplementary material.

Supplementary file1 (DOCX 17 KB)
