# Supplementary material for: Residual myocardial hyperemia in regadenoson stress/rest quantitative perfusion cardiac magnetic resonance
Source: Radiol Med. 2025 Aug 23;130(11):1777–85. doi: 10.1007/s11547-025-02062-3 (PMC12605555; doi:10.1007/s11547-025-02062-3)
Supplement: Supplementary file 1 — Supplementary file1 (DOCX 17 KB) [file 11547_2025_2062_MOESM1_ESM.docx]

**Supplemental table 1**. Individual MBF/MPR values.

| **Individual** | **MBF_rest_** | **MBF_stress_** | **MBF_recovery_** | **MPR_stress/rest_** | **MPR_stress/recovery_** |
| --- | --- | --- | --- | --- | --- |
| 1 | 0,85 | 2,96 | 1,06 | 3,48 | 2,8 |
| 2 | 0,65 | 1,69 | 1,69 | 2,61 | 2,48 |
| 3 | 1,52 | 2,33 | 1,52 | 1,52 | 1,54 |
| 4 | 0,78 | 2,08 | 0,94 | 2,16 | 2,22 |
| 5 | 0,86 | 1,48 | 0,86 | 1,73 | 1,72 |
| 6 | 0,7 | 1,32 | 0,74 | 1,88 | 1,77 |
| 7 | 1,19 | 1,44 | 1,21 | 1,21 | 1,19 |
| 8 | 0,61 | 2,28 | 0,74 | 3,72 | 3,09 |
| 9 | 0,85 | 2,32 | 0,85 | 2,73 | 2,74 |
| 10 | 0,83 | 2,53 | 0,85 | 3,04 | 2,99 |
| 11 | 0,58 | 2,17 | 0,58 | 3,28 | 3,73 |
| 12 | 0,75 | 1,55 | 0,61 | 2,06 | 2,53 |
| 13 | 0,98 | 2,89 | 1,22 | 2,95 | 2,37 |
| 14 | 0,93 | 2,6 | 0,88 | 2,79 | 2,94 |
| 15 | 0,56 | 1,27 | 0,73 | 2,26 | 1,74 |
| 16 | 0,87 | 2,84 | 1,01 | 3,25 | 2,82 |
| 17 | 0,68 | 2,97 | 0,65 | 4,39 | 4,53 |
| 18 | 0,63 | 1,29 | 0,59 | 2,06 | 2,19 |
| 19 | 0,97 | 2,87 | 0,94 | 2,96 | 3,07 |
| 20 | 0,82 | 2,18 | 1,03 | 2,64 | 2,11 |
| 21 | 0,55 | 1,52 | 0,55 | 2,4 | 2,75 |
| 22 | 0,83 | 1,12 | 0,76 | 1,36 | 1,48 |
| 23 | 0,64 | 1,64 | 0,69 | 2,51 | 2,34 |
| 24 | 0,98 | 2,81 | 1,12 | 2,87 | 2,51 |
| 25 | 0,88 | 2,81 | 0,98 | 3,18 | 2,87 |
| 26 | 0,83 | 1,6 | 0,83 | 2,04 | 1,92 |
| 27 | 0,86 | 2,62 | 0,93 | 3,05 | 2,8 |
| 28 | 1,01 | 2,02 | 1,12 | 2,01 | 1,81 |
| 29 | 1,02 | 2,69 | 1,4 | 2,63 | 1,92 |
| 30 | 0,61 | 1,25 | 0,68 | 2,06 | 1,85 |

**Supplemental table 2**. Repeated measures within subjects of myocardial blood flow (MBF) and myocardial perfusion reserve (MPR) per myocardial segment.

| **Myocardial segment** | **Measurement** | **Mean difference** | **Std. Error** | **P-value** | **95% CI** |
| --- | --- | --- | --- | --- | --- |
| Basal anterior | MBF | 0.13 | 0.05 | 0.005 | 0.04 - 0.22 |
|  | MPR | -0.22 | 0.08 | 0.008 | -0.38 - -0.06 |
| Basal anteroseptal | MBF | 0.09 | 0.049 | 0.054 | -0.01 - 0.19 |
|  | MPR | -0.15 | 0.08 | 0.067 | -0.31 - 0.01 |
| Basal inferoseptal | MBF | 0.11 | 0.03 | 0.002 | 0.04 - 0.17 |
|  | MPR | -0.24 | 0.10 | 0.020 | -0.45 - -0.04 |
| Basal inferior | MBF | 0.05 | 0.04 | 0.169 | -0.02 - 0.12 |
|  | MPR | -0.05 | 0.11 | 0.644 | -0.26 - 0.16 |
| Basal inferolateral | MBF | 0.03 | 0.03 | 0.417 | -0.04 - 0.09 |
|  | MPR | 0.04 | 0.09 | 0.695 | -0.15 - 0.23 |
| Basal anterolateral | MBF | 0.11 | 0.04 | 0.004 | 0.03 - 0.18 |
|  | MPR | -0.29 | 0.15 | 0.059 | -0.59 - 0.01 |
| Mid anterior | MBF | 0.14 | 0.04 | 0.001 | 0.06 - 0.22 |
|  | MPR | -0.29 | 0.06 | <0.001 | -0.42 - -0.16 |
| Mid anteroseptal | MBF | 0.11 | 0.04 | 0.016 | 0.02 - 0.19 |
|  | MPR | -0.19 | 0.07 | 0.008 | -0.33 - -0.05 |
| Mid inferoseptal | MBF | 0.09 | 0.04 | 0.024 | 0.01 - 0.17 |
|  | MPR | -0.13 | 0.06 | 0.037 | -0.25 - -0.02 |
| Mid inferior | MBF | 0.09 | 0.04 | 0.019 | 0.016 - 0.17 |
|  | MPR | -0.1 | 0.07 | 0.112 | -0.25 - 0.03 |
| Mid inferolateral | MBF | 0.07 | 0.04 | 0.078 | -0.01 - 0.15 |
|  | MPR | -0.02 | 0.08 | 0.775 | -0.18 - 0.13 |
| Mid anterolateral | MBF | 0.09 | 0.04 | 0.019 | 0.02 - 0.17 |
|  | MPR | -0.12 | 0.06 | 0.036 | -0.23 - -0.01 |
| Apical anterior | MBF | 0.14 | 0.05 | 0.005 | 0.04 - 0.23 |
|  | MPR | -0.27 | 0.09 | 0.003 | -0.45 - -0.09 |
| Apical septal | MBF | 0.13 | 0.04 | 0.002 | 0.04 - 0.21 |
|  | MPR | 0.10 | 0.04 | 0.027 | 0.01 - 0.19 |
| Apical inferior | MBF | 0.1 | 0.04 | 0.019 | 0.02 - 0.17 |
|  | MPR | -0.06 | 0.12 | 0.639 | -0.29 - 0.18 |
| Apical lateral | MBF | 0.13 | 0.05 | 0.016 | 0.02 - 0.24 |
|  | MPR | -0.19 | 0.10 | 0.059 | -0.39 - 0.01 |

Note. Std. Error: standard error; CI: confidence interval.
